# Pharmaceutical care of rituximab in the treatment of children with refractory anti-NMDAR encephalitis: A case report

**DOI:** 10.1097/MD.0000000000032843

**Published:** 2023-02-03

**Authors:** Haiyan Wu, Xiaoyan Xu, Qixuan Ding, Shuangfei Zhu, Qiaozhen Zheng, Shanshan Ding, Jiyao Li, Hongyang Zhao

**Affiliations:** a Department of Pharmacy, Central Hospital Affiliated to Shandong First Medical University, Jinan, China; b School of Pharmacy, Shandong First Medical University, Jinan, China; c School of Clinical Pharmacy, Shandong First Medical University, Jinan, China; d Department of Pharmacy, Liaocheng People’s Hospital, Liaocheng, China; e Department of Pediatrics, Central Hospital Affiliated to Shandong First Medical University, Jinan, China.

**Keywords:** anti-infective drugs, anti-NMDAR encephalitis, off-label use, pharmaceutical care, rituximab

## Abstract

Anti-*N*-methyl-d-aspartate receptor (NMDAR) encephalitis is a rare disease of nervous system, which is mediated by autoimmune mechanisms. The treatment of anti-NMDAR encephalitis includes Immunotherapy, symptomatic and supportive treatment for seizures and psychiatric symptoms. There are many kinds of drugs, so drug treatment management and pharmaceutical care for children are particularly important. At present, there are few reports on pharmaceutical care for children with this disease. Clinical pharmacists participated in the pharmaceutical care of a child with refractory anti-NMDAR encephalitis treated with rituximab, conducted drug treatment management on the dosage, administration method, complications and other aspects of off-label use of rituximab, combined with the children’s clinical manifestations, inflammatory indicators, pathogenic detection, blood concentration, liver and kidney functions, drug interactions and other factors. The treatment plan of anti-infective drugs shall be adjusted, and attention shall be paid to whether there are adverse reactions during the treatment.

**Patient concerns:**

A 4-year-old girl presented with epileptic seizure, intermittent recurrent fever, high inflammatory markers, abnormal psychiatric function/cognitive impairment, language disorder, consciousness disturbance, and movement disorder/involuntary movement.

**Diagnosis:**

Refractory anti-NMDAR encephalitis.

**Interventions:**

The patient was given first-line (3 rounds of methylprednisolone pulse therapy and gamma globulin) and second-line (rituximab) immunotherapy. On the advice of a clinical pharmacist, the patient wasn’t given Advanced antibacterial agents (voriconazole, vancomycin) therapy. On the 41st day of admission, the patient’s temperature and inflammatory indicators were normal, CD19^+^ B cells were reduced to 0.

**Outcomes:**

The patient consciousness level, cognition and orientation were gradually improved, mental disorder was improved, involuntary movement was obviously controlled, no seizure occurred again, and the patient was discharged with stable condition.

**Lessons:**

Clinical pharmacists ensure the safety, effectiveness and economy of patients’ medication by carrying out the whole process of individualized drug treatment management and care for patients.

## 1. Introduction

Autoimmune encephalitis is a rare disease of nervous system, which is mediated by autoimmune mechanisms. Anti-*N*-methyl-d-aspartate receptor (NMDAR) encephalitis was first reported in 2007.^[1]^ At present, it is the most common autoimmune encephalitis, accounting for 54% to 80% of autoimmune encephalitis.^[2]^ It is mainly seen in children and adolescents, and more common in women than in men. Nearly 60% of adult patients with anti NMDAR encephalitis are accompanied by tumors, and tumor resection is an important method to treat the disease. However, only 6% of children with Anti-NMDAR encephalitis accompanied by tumors, and immunotherapy is the main treatment method.^[3]^ Immunotherapy can improve the prognosis of NMDAR encephalitis and reduce the risk of recurrence, especially early administration.^[4–9]^ The treatment of anti-NMDAR encephalitis also includes symptomatic and supportive treatment for seizures and psychiatric symptoms.^[10]^ There are many kinds of drugs, so drug treatment management and pharmaceutical care for children are particularly important. At present, there are few reports on pharmaceutical care for children with this disease. This article analyzes the treatment process of a child with rituximab-refractory anti-NMDAR encephalitis, hoping to provide reference for the drug treatment management and service work of such severe and refractory anti-NMDAR encephalitis children.

## 2. Case report

A 4-year-old girl was admitted to the hospital on June 4, 2022 due to “intermittent fever for 7 days and anorexia for 2 days.” Seven days ago, the patient was T38.4°C, and had no other symptoms such as cough and vomiting. Two days ago, she had poor appetite and poor spirit, and was treated with antibacterial drugs and antiviral drugs, but the effect was poor. The patient had been healthy.

### 2.1. Admission examination

T37.1°C, normal spirit, stable breathing, good skin elasticity, no rash, ruddy lips, pharyngeal congestion, II degree tonsil enlargement, a purulent white spot on the surface of the left tonsil, and no resistance to neck. The breath sounds of both lungs were coarse, and no obvious lung rales were heard. The heart rate was 104 beats/min, the rhythm was normal, and no pathological murmur was heard. The abdomen was soft and nontender, and hepatosplenomegaly was not palpable. There were no abnormalities in the spine or limbs.

### 2.2. Laboratory examination

2022-05-28 blood routine examination + C-reactive protein (CRP) (external hospital): white blood cell (WBC) 8.0 × 10^9^/L, neutrophils 59%, lymphocytes 32.3%, CRP < 5 mg/L.

2022-06-04 Blood cell analysis + CRP (emergency): WBCs 8.95 × 10^9^/L, neutrophils 69.3%, lymphocytes 23.1%, CRP < 0.50 mg/L.

## 3. Treatment process

On day 2 to 6 of admission, the child gradually developed clinical symptoms such as gibberish, abnormal mental behavior, seizures, restlessness and involuntary movement. The patient was transferred from the pediatric respiratory department to the pediatric neurology department. Complete auxiliary examinations: blood routine + CRP, procalcitonin (PCT), erythrocyte sedimentation rate, abnormal WBC morphology, liver function, kidney function, blood ions, blood plasma ammonia, cerebrospinal fluid biochemistry, EB virus, ASO, blood culture, sputum culture, T-SPOT, G test, GM test, brain computed tomography and head magnetic resonance imaging showed no obvious abnormalities. The patient had a high WBC count in cerebrospinal fluid (WBC 9 × 10^6^/L), abnormal electroencephalogram (slow wave activity during waking and sleeping), positive NMDAR-R-Ab in cerebrospinal fluid (1:320, d6), and a modified Rankin score of 4-5. The patient was diagnosed as severe autoimmune encephalitis (anti NMDAR encephalitis). No obvious abnormality was found in chest and abdomen computed tomography and blood tumor markers. Ceftriaxone was given for anti-infection and first-line immunotherapy (methylprednisolone pulse therapy + gamma globulin) was given for anti-NMDAR encephalitis. On the 10th day of admission, the patient had convulsions again and was treated with sodium valproate. On the 24th day after admission, the children were given 3 rounds of first-line immunotherapy (methylprednisolone pulse therapy + gamma globulin), but the condition was still not under control. The second line immunotherapy was rituximab (once a week, on the 24th/31st/37th day). See Table 1 for main drug treatment.

**Table 1:** Main drug treatment during hospitalization.

Classification of drug therapy	Drug use	
Immunotherapy drugs	Methylprednisolone	Day 6–8/day 13–15/day 20–23
	Gamma globulin	Day 3–5/day 18–19/day 38–39
	Rituximab	Day 24/day 31/day 37
Anti infective drugs	Cefatriaxone	Day 2–27
	Cefoperazone sulbactam sodium	Day 28–42
	Erythromycin	Day 28–33

Drugs for mental behavior disorders and sleep disorders: risperidone, haloperidol, diazepam, clonazepam, melatonin tablets were given successively to treat mental behavior disorders and improve sleep disorders, and diazepam, midazolam, and chloral hydrate were given intermittently for sedation.

On the 28th day of admission, the child had intermittent fever, WBC 17.09 × 10^9^/L, CRP 40.23 mg/L, PCT 0.668 ng/mL, Mycoplasma pneumoniae IgM antibody 1.45 COI, Mycoplasma pneumoniae IgG antibody 104 AU/mL. Erythromycin combined with cefoperazone sulbactam sodium was given. On the 31st day of admission, the patient still had intermittent fever, WBC 12.36 × 10^9^/L, CRP 42.3 5mg/L, PCT 0.859ng/mL. The clinical pharmacist suggested that the drug should not be added temporarily, and the DNA of mycoplasma pneumoniae should be tested and meanwhile monitor various inflammatory indicators and bacterial and fungal culture. On the 33rd day of admission, the child’s temperature was 36.7 to 37.9°C, WBC and CRP decreased, Mycoplasma pneumoniae DNA was negative, and liver enzymes (alanine aminotransferase 133.7 × U/L, aspartate aminotransferase 76.1 × U/L) increased. The clinical pharmacist suggested stopping erythromycin. On the 37th day of admission, the patient’s temperature and inflammatory indicators (WBC7.07 × 10^9^/L, CRP 0.86 mg/L) were normal, liver enzymes (alanine aminotransferase 62.4 U/L, aspartate aminotransferase 40.9 U/L) decreased significantly, and the patient’s condition improved. The third rituximab treatment was given as planned. On the 41st day of admission, the patient’s temperature and inflammatory indicators were normal, CD19^+^ B cells were reduced to 0, consciousness level, cognition and orientation were gradually improved, mental disorder was improved, involuntary movement was obviously controlled, no seizure occurred again, and the patient was discharged with stable condition.

## 4. Pharmaceutical care, analysis, and discussion

### 4.1. Drug treatment management of rituximab in anti NMDAR encephalitis

The diagnosis of children’s autoimmune encephalitis is clear, and first-line immunotherapy is given according to the consensus of experts. However, after 3 rounds of first-line immunotherapy (methylprednisolone pulse therapy + gamma globulin), the condition of children has not improved significantly. How to choose immunotherapy?

Rituximab is a human mouse chimeric monoclonal antibody that can specifically bind to transmembrane antigen CD20 and selectively inhibit the function of B cells.^[11]^ It is a second-line immune drug against NMDAR encephalitis and is mainly used for severe patients who have not improved significantly after two or more first-line immunotherapy.^[10]^ International consensus recommendation on the treatment of children’s NMDAR antibody encephalitis^[12]^: the effect of second-line treatment is better than that of first-line treatment for patients who do not improve after 2 weeks of first-line treatment or more (especially severe patients). Early use of rituximab is more beneficial for children than delayed use. In a study conducted by Professor Li’s team,^[13]^ 36 children with anti-NMDAR encephalitis were investigated (29 received first-line treatment and 7 received rituximab after first-line treatment). After 1-year follow-up, 6 children relapsed in the first-line treatment group, while there was no recurrence in the rituximab group.

The patient is a severe refractory anti-NMDAR encephalitis. After 18 days of first-line treatment, there is no significant improvement in her condition, which is consistent with the indications of rituximab. After consultation with clinical pharmacists, off-label drug was record, and the family members were informed and agreed to use the drug.

### 4.2. Administration plan and medication monitoring administration plan

#### 4.2.1. Dosing regimens

The current regimens of rituximab for the treatment of anti-NMDAR are diverse, as detailed in Table 2.

**Table 2:** Dosing regimens.

Reference basis	Administration plan
Expert consensus on diagnosis and treatment of autoimmune encephalitis in China (2022 version)	• The routine regimen was 375 mg/m^2^. The drug was given once a week for 3 to 4 times in total. • Dose reduction protocol: • The total dose was 600 mg (100 mg on the first day and 500 mg on the second day). • Total dose 400 mg (100 mg/wk, 4 times)^[10,14,15]^
International consensus recommendations on the treatment of NMDAR antibody encephalitis in children (2021)	• 500 to 1000 mg (500 mg for patients less than 40 kg, 1000 mg for patients more than 40 kg), given twice with an interval of 2 weeks; • 375 to 750 mg/m^2^ (maximum 1 g) administered twice with an interval of 2 weeks; • 375 mg/m^2^ (maximum 1 g) for 4 weeks

NMDAR = *N*-methyl-d-aspartate receptor.

The treatment plan for this patient is 375 mg/m^2^, intravenous drip, once a week, according to the level of CD20 positive B cells in peripheral blood, a total of 3 to 4 times, until the peripheral blood CD20 cells are cleared.

#### 4.2.2. Medication monitoring

There were many adverse reactions of rituximab, especially transfusion related adverse reactions, neutropenia, hypoproteinemia. For bedridden patients, there may be secondary serious infection, such as pneumocystis carinii pneumonia. Clinical pharmacists and clinicians jointly formulate drug treatment management, give paracetamol and prednisolone before infusion to reduce the occurrence of adverse reactions, and give compound sulfamethoxazole tablets (3 days a week) to prevent pneumocystis carinii pneumonia. At the same time, pay attention to the dripping speed (gradually adjust the pump speed to 10 mL/h–25 mL/h–50 mL/h–75 mL/h, and increase the speed once every half an hour), monitor vital signs (monitor vital signs every 15 minutes in the first hour, once every half an hour in the second hour, and then once every hour until drug withdrawal), liver and kidney functions, electrolytes, blood routine, T/B cell subsets, immunoglobulins, etiology, etc., and discontinue the drug in case of discomfort or abnormal clinical manifestations. Treat symptomatically to ensure the drug safety of patients.

After 3 times of administration, the CD19^+^ B cells of the patient decreased to 0, so the fourth administration was suspended, and no adverse reactions such as infusion reaction occurred during the period. In this study, there was no significant improvement in the patients following treatment with corticosteroids combined with IVIG. After treatment with rituximab, the nervous system symptoms and levels of CD19^+^ B-cells were significantly improved. The patient showed obvious B-cell depletion within 3 weeks after the first infusion, and the CD19^+^ B-cell count was 0. This finding is consistent with those observed in studies conducted by Professor Li’s team.^[13]^

### 4.3. Anti-infective drug treatment management

On the 31st day of admission, the child still had recurrent fever, high inflammatory indicators (WBC12.36 × 10^9^/L, CRP 42.35 mg/L, PCT 0.859 ng/mL), and pulmonary infection still existed in auscultation and chest radiograph. It does not rule out the infection of gram-positive bacteria. At the same time, the patient’s immunity is low, the use of antibacterial drugs is long, and there is a possibility of fungal infection. Should vancomycin and fluconazole be added?

The clinical pharmacist consulted and checked the pathogen detection: G test, GM test, blood culture, sputum culture, cerebrospinal fluid bacterial culture and their identification + Cryptococcus smear examination were negative, and there was no indication for adding vancomycin and fluconazole temporarily. Combined with the clinical manifestations, etiology and inflammatory indicators of the children, the analysis was carried out as follows: severe anti NMDAR encephalitis itself may lead to autonomic nervous dysfunction, followed by temperature regulation dysfunction and temperature fluctuation; immune diseases may also have high CRP; the daily temperature rise usually occurs when the children are obviously restless. Calm down and cool down with ice blanket for 1 to 2 hours, and the temperature can quickly drop to normal. Therefore, involuntary movement may increase when the children are restless, and the skeletal muscle may produce heat, leading to temperature fluctuations; the child still had cough and phlegm from time to time, and pulmonary auscultation could be heard with phlegm rale. Chest X-ray showed double pneumonia changes with high inflammatory indicators, which did not rule out that the infection was not completely controlled. Studies have reported that the common pathogens of autoimmune encephalitis are herpes simplex virus or Japanese encephalitis virus,^[16]^ and mycoplasma has also been detected in patients with autoimmune encephalitis.^[17,18]^ In this case, only mycoplasma pneumoniae antibody was positive, and the rest were negative. It does not rule out that the current fever and high inflammatory indicators are caused by mycoplasma pneumoniae infection.

To sum up, the clinical pharmacist suggested that vancomycin and fluconazole should not be used for the time being, and that Mycoplasma pneumoniae DNA testing should be carried out to determine whether there is Mycoplasma pneumoniae infection at present. At the same time, inflammatory indicators and bacterial fungal culture should be monitored to determine the next step of anti-infection drug selection. The test results showed that Mycoplasma pneumoniae DNA was negative and liver enzyme was elevated. The clinical pharmacist considered that there was no Mycoplasma pneumoniae infection at present, and the elevation of liver enzyme could not be ruled out as an adverse reaction of erythromycin, so it was recommended to stop erythromycin. On the 38th day of admission, the temperature and inflammatory indicators were normal, and the patient’s condition gradually improved.

Autoimmune encephalitis is often accompanied by epileptic seizures. And attention should be paid to the selection of antibacterials. Patients who use sodium valproate are prohibited from using meropenem. Drug instructions, the detailed rules for the evaluation of the clinical application of carbapenems and the literature all pointed out that there was a complex interaction between carbapenems and sodium valproate, which would reduce the blood concentration of valproic acid and lead to seizure recurrence. The two should not be used together. Carbapenems can reduce the blood concentration of sodium valproate by about 60% to 100% within 2 days. Meropenem has an impact on the absorption, distribution, metabolism and excretion of sodium valproate. Through the effects of different links, the blood concentration of sodium valproate will eventually be significantly reduced, and there is no way to control it by increasing the dosage of sodium valproate.^[19–21]^ There are literature reports that meropenem has successfully treated cases of valproic acid overdose poisoning,^[22]^ so meropenem should not be used in combination with sodium valproate.

## 5. Summary

The acute stage of anti-NMDAR encephalitis is severe, with a mortality rate of about 5%, and about 15% of patients may relapse.^[3]^ Rituximab can improve the prognosis of patients with ineffective first-line treatment, reduce the risk of recurrence, and early use is more conducive to the prognosis of children.^[12]^ This patient is a severe refractory anti NMDAR encephalitis. It is necessary to give rituximab as early as possible after the first-line drug treatment fails.

Persistent autonomy disorder, fever and deterioration of psychological state are difficult to manage. It is usually impossible to distinguish the primary disease from the hospital complications, resulting in the patient receiving long-term sedatives, neuromuscular blockers, empirical antibiotics, neuroactive and psychoactive drugs.^[23]^ The patient has a long fever time, high inflammatory indicators, and intense involuntary exercise. It is difficult to distinguish between central noninfectious fever and infectious fever caused by the primary disease. Owing to many drugs are used, clinicians and pharmacists must improve their understanding of diseases and drugs, select appropriate drugs, and be alert to drug interactions and adverse reactions.

Clinical pharmacists actively participate in the whole process of patients’ treatment, and assist physicians in the formulation and implementation of treatment plans by searching relevant disease diagnosis and treatment guidelines and drug treatment manuals. To achieve the therapeutic purpose, reduce the use of drugs, reduce the occurrence of adverse reactions, and ensure the effectiveness and safety of drug use for patients.

## Author contributions

**Conceptualization:** Haiyan Wu.

**Data curation:** Haiyan Wu, Qixuan Ding, Shuangfei Zhu, Qiaozhen Zheng

**Formal analysis:** Haiyan Wu, Shanshan Ding, Jiyao Li.

**Investigation:** Haiyan Wu, Qixuan Ding, Shuangfei Zhu, Qiaozhen Zheng

**Project administration:** Haiyan Wu, Xiaoyan Xu, Hongyang Zhao.

**Supervision:** Hongyang Zhao.

**Writing – original draft:** Haiyan Wu

**Writing – review & editing:** Haiyan Wu, Xiaoyan Xu, Hongyang Zhao, Shanshan Ding, Jiyao Li.
